# COVID-19 and Sudden Sensorineural Hearing Loss: A Systematic Review

**DOI:** 10.3389/fneur.2022.883749

**Published:** 2022-04-28

**Authors:** Xiangming Meng, Jing Wang, Jian Sun, Kangxu Zhu

**Affiliations:** ^1^Department of Otolaryngology, Wuxi Huishan District People's Hospital, Wuxi, China; ^2^Department of Otolaryngology, Huadong Sanatorium, Wuxi, China; ^3^Department of Rheumatology and Immunology, Affiliated Hospital of Jiangnan University, Wuxi, China

**Keywords:** COVID-19, SARS-CoV-2, sudden sensorineural hearing loss, inner ear, incidence, glucocorticoids

## Abstract

A growing body of evidence suggests that patients with the 2019 Coronavirus disease (COVID-19) have a risk of developing sudden sensorineural hearing loss (SSNHL). The pathogenesis of COVID-19-related SSNHL remains unclear. This systematic review examined whether COVID-19 causes an increased incidence of SSNHL and the clinical characteristics of patients with COVID-19-related SSNHL according to the Preferred Reporting Items for Systematic reviews and Meta-Analyses (PRISMA) guidelines. SSNHL usually developed between a few days and 2 months after the diagnosis of COVID-19, and a proportion of patients developed it before the diagnosis of COVID-19. The literature is inconsistent regarding whether COVID-19 causes an increased incidence of SSNHL, and this matter remains unclear. This review included 23 patients with COVID-19-related SSNHL, all adult patients with an average age of 43.1 years. Of these patients, 60.9% had accompanying tinnitus symptoms. Glucocorticoids are the preferred medication to treat COVID-19-related SSNHL. Intratympanic administration may be considered to reduce the side effects of the drug. Hearing tests are suggested when hearing loss is suspected in COVID-19 individuals, and if SSNHL is detected, prompt and aggressive treatment is vital. Large-scale, multicenter research on the pathophysiology, treatment, and prognosis of COVID-19- related SSNHL should be conducted in the future.

## Introduction

A novel infectious disease, 2019 Coronavirus disease (COVID-19) is caused by severe acute respiratory syndrome coronavirus 2 (SARS-CoV-2), which is a highly transmissible and pathogenic human coronavirus (1). Scientific and clinical evidence suggested that COVID-19 can affect multiple organ systems (2). SARS-CoV-2 can also invade the central and peripheral nervous system to cause various neurological diseases (3).

Sudden sensorineural hearing loss (SSNHL) is defined as the sudden onset of sensorineural hearing loss (SNHL) without an identifiable etiology, with at least three consecutive frequency losses ≥ 30 dB within 72 h (4). The annual incidence of SSNHL varies between 5 and 27 cases per 100,000 persons (5). The cause of SSNHL is unclear. However, the possible causes include viral infection, circulatory abnormalities, autoimmune disease, labyrinthine membrane rupture, and central nervous system (CNS) anomalies (5, 6).

Strong evidence suggests that viral infection is a cause of SSNHL (7). It may be related to direct viral access to the labyrinth or the cochlear nerve, reactivation of latent virus inside spiral ganglia, and immunoregulation of systemic viral infections (7). Lassa fever, mumps, adenovirus, and other viruses have caused SSNHL (8–10). Growing data indicate that patients with COVID-19 are prone to SSNHL (7, 11–13). However, many aspects of COVID-19-related SSNHL have remained unclear.

The purpose of this review was to evaluate the impact of COVID-19 on the incidence of SSNHL and to provide an understanding of the clinical characteristics of COVID-19-related SSNHL.

## Methods

A systematic review was conducted based on the Preferred Reporting Items for Systematic reviews and Meta-Analyses (PRISMA) guidelines (14). Ethics committee approval was not required for this literature review.

### Search Strategy

We systematically searched literature databases, including PubMed, Scopus, Web of Science, and Embase, using variations of the descriptors for “COVID-19,” “SARS-CoV-2,” “SSNHL” with AND/OR. Searches were not restricted to studies by date, publication status, or language. The retrieval scheme was mainly based on a combination of medical subject headings (MeSH) terms and free words. The final search was performed on February 22, 2022. Two reviewers (JW and KZ) independently assessed the eligibility of the studies and extracted the data.

### Inclusion/Exclusion Criteria for Study Selection

The inclusion criteria were original studies or case reports, human studies, and studies written in English. Included patients required confirmation of COVID-19 by polymerase chain reaction testing or antibody testing. Irrelevant articles, non-English language articles, review papers, letters without data, commentary, studies of SSNHL after COVID-19 vaccine, and abstracts without full text were excluded.

### Data Extraction

Two reviewers (JW and KZ) extracted data using a well-designed Excel (Microsoft Inc., USA) spreadsheet. The following data were included: authors, year of publication, the country in which the study was conducted, study design, study purpose, assessment period, main results, conclusions, sample size, age and gender, time of onset, side affected, symptoms, audiological assessment, treatments, and treatment outcome.

### Assessment of Risk of Bias

The two reviewers (JW and KZ) independently scored each study. The quality of studies for inclusion in the review was assessed using the National Institutes of Health quality assessment tool for case series studies, as well as observational cohort and cross-sectional studies (10). Based on the rating of each study, its quality rating can be divided into three categories: good, fair, and poor. Any disagreement was resolved by consensus through the authors' discussions.

## Results

### Search Outcome

**Figure 1:**
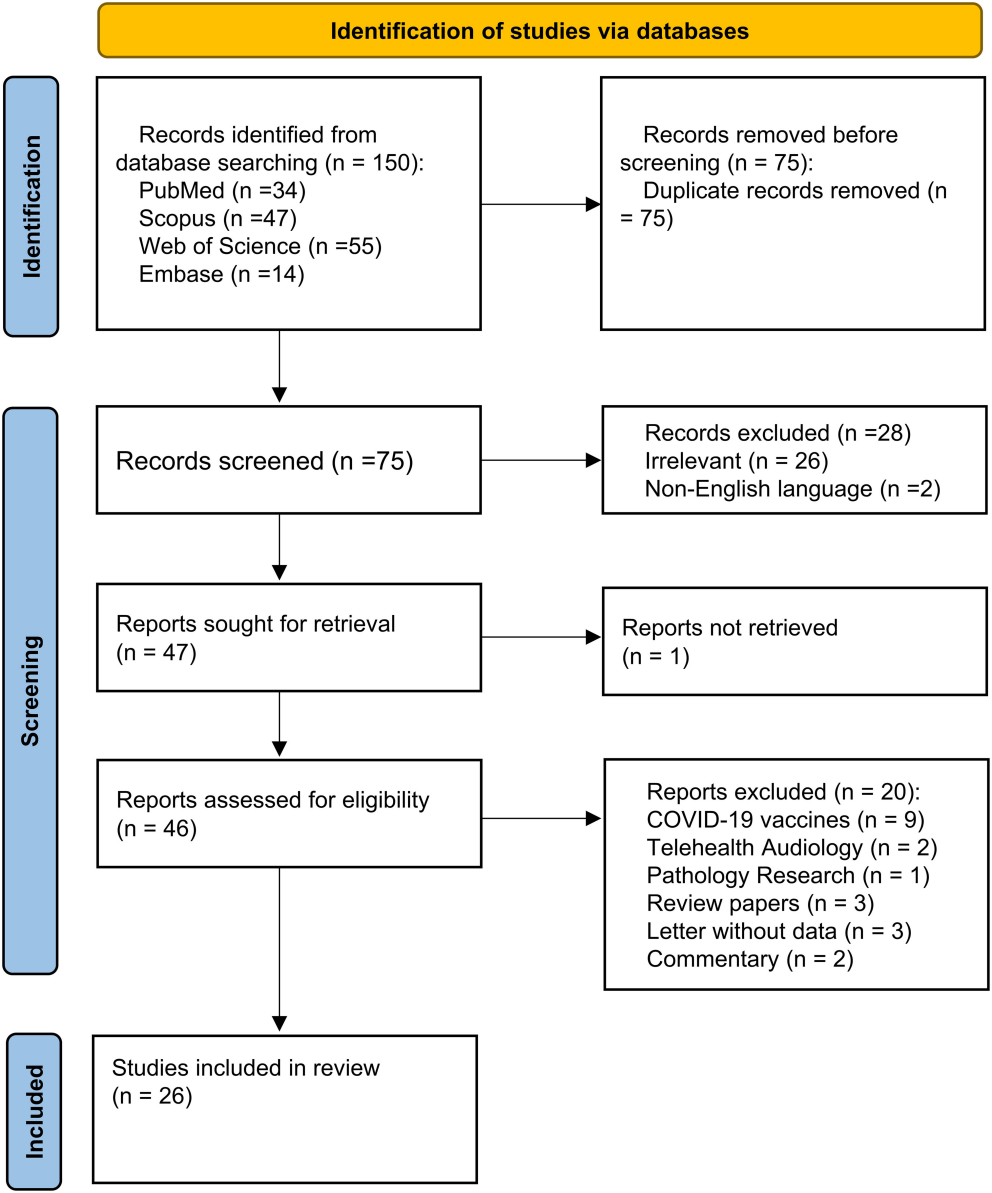
Flow chart showing the process of literature screening.

There were eventually 26 studies included in this systematic review. Ten papers worldwide have investigated various aspects of whether COVID-19 contributes to the increased incidence of SSNHL. These studies are summarized in Table 1. Sixteen articles studied the clinical characteristics and therapeutic outcomes of COVID-19-related SSNHL. Of these 16 studies, 13 were case reports, and 3 were case series. The clinical characteristics of the patients with COVID-19-related SSNHL are synthesized in Table 2. A flowchart of the literature search is presented in Figure 1.

**Table 1:** A summary of studies on the relationship between 2019 Coronavirus disease (COVID-19) and the incidence of sudden sensorineural hearing loss (SSNHL).

**No**.	**References**	**Country**	**Study design**	**Purposes**	**Assessment period**	**Main results**	**Conclusions**	**Risk of bias**
1	Swain et al. (15)	India	Prospective study	To investigate the incidence of HL in COVID-19 patients after hospital discharge.	March to August, 2020	Of the 472 COVID-19 patients, 28 presented with HL, 17 of whom were SSNHL.	COVID-19 infection may have harmful effects on the IE.	Poor
2	Thrane et al. (16)	Denmark	Retrospective study	To investigate self-reported hearing symptoms among COVID-19 patients.	April 22 to November 5, 2020	Of the 225 respondents with chemosensory loss, 24 (10.7%) reported concomitant HL.	A significant proportion of COVID-19 patients presented with concomitant audiological symptoms.	Fair
3	Parrino et al. (17)	Italy	Retrospective study	To assess the impact of COVID-19 on the incidence of SSNHL and vestibular disease.	March 1, 2019 to February 29, 2020	The overall incidence of SSNHL and combined acute cochlear-vestibular involvement was significantly higher during COVID-19 than in previous periods.	SSNHL appeared worse in terms of PTA during the pandemic.	Good
4	Fidan et al. (18)	Turkey	Retrospective study	To measure the incidence of SSNHL presenting at otolaryngology clinic during the COVID-19 and pre-pandemic period.	April 1 and September 30, 2020	68 patients with SSNHL in 2020, 41 patients with SSNHL in 2019.	The incidence of SSNHL increased during the COVID-19 pandemic compared with the same period of the previous year.	Poor
5	Jin et al. (19)	China	Retrospective study	To evaluate the impact of COVID-19 on otolaryngological common diseases.	February to April 2020	SSNHL increased in outpatient otolaryngology clinics.	COVID-19 may lead to an increase in patients with SSNHL and tinnitus.	Fair
6	Chari et al. (20)	USA	Retrospective study	To assess the impact of the viral outbreak on clinical presentations of SSNHL at a single center.	March 15 to May 31, 2020	The number of SSNHL patients declined during the COVID-19 outbreak.	COVID-19 does not appear to increase the risk of developing SSNHL significantly.	Fair
7	Hafrén et al. (21)	Finland	Retrospective study	To investigate changes in the incidence of SSNHL during the lockdown.	January 1, 2017 to August 31, 2020	No change in the incidence of SSNHL	Respiratory pathogens might be an etiology of Bell's palsy.	Poor
8	Aslan et al. (22)	Turkey	Retrospective study	To evaluate the relationship of SSNHL and Bell's palsy with COVID-19.	April 2020 to April 2021	No significant differences in the incidence of SSNHL and Bell's palsy compared to years without the pandemic.	No association was observed between cases of SSNHL and Bell's palsy and COVID-19.	Fair
9	Van Rijssen et al. (23)	Dutch	Prospective study	To investigate the incidence of COVID-19 in patients with SSNHL.	November 2020 to March 2021	No COVID-19 positive patients were detected among the 25 SSNHL patients.	No significant relationship between SSNHL and COVID-19.	Good
10	Doweck et al. (24)	Israel	Cross-sectional study	To investigate the incidence of SSNHL during the COVID-19 pandemic and its association with lockdowns.	2020	Significant decrease in SSNHL during the COVID-19 pandemic.	It might be associated with social distancing, lockdowns.	Good

*COVID-19, 2019 Coronavirus disease; HL, hearing loss; SSNHL, Sudden sensorineural hearing loss; IE, Inner ear; PTA, Pure-tone thresholds*.

**Table 2:** Clinical characteristics and treatment outcomes of patients with COVID-19-related SSNHL.

**No**.	**References**	**Country**	**Study design**	**Sample size**	**Age, gender**	**Time of onset**	**Side affected**	**Symptoms**	**Audiological assessment**	**Treatments**	**Treatment outcome**	**Risk of bias**
1	Ozer et al. (25)	Turkey	Case report	1	62, F	2 days before COVID-19	Left	HL, Peripheral facial paralysis	PTA, ABR, ENoG	Oral corticosteroids	Partial recovery	Good
2	Degen et al. (12)	Germany	Case report	1	60, M	13 days after COVID-19	Bilaterally	HL, Tinnitus	ABR	IM corticosteroids, CI	No improvement	Fair
3	Chern et al. (7)	USA	Case report	1	18, F	7 weeks before COVID-19	Bilaterally	HL, Vertigo	PTA, WRS	Oral corticosteroids, IM corticosteroids	Partial recovery	Good
4	Gunay et al. (26)	Turkey	Case report	1	23, F	3 days before COVID-19	Bilaterally	HL, Ear pain	PTA, Tympanometry	Oral corticosteroids	Partial recovery	Fair
5	Beckers et al. (27)	Belgium	Case report	1	53, M	12 days before COVID-19	Right	HL	PTA, VHIT	Intravenous corticosteroids, Oral corticosteroids	Partial recovery	Fair
6	Gerstacker et al. (28)	Germany	Case report	1	38, M	About 8 weeks after COVID-19	Bilaterally	HL, Tinnitus, Vertigo	PTA, TFT, OAE, ABR, VNG	Oral corticosteroids, IM corticosteroids, CI, Hearing aid	No improvement	Good
7	Guigou et al. (29)	France	Case report	1	29, M	10 days before COVID-19	Bilaterally	HL	PTA	Oral corticosteroids	Complete recovery	Fair
8	Koumpa et al. (30)	UK	Case report	1	45, M	About 7 weeks after COVID-19	Left	HL, Tinnitus	PTA, TFT	Oral corticosteroids, IM corticosteroids	Partial recovery	Good
9	Lamounier et al. (31)	Brazil	Case report	1	67, F	About 3 weeks after COVID-19	Right	HL, Tinnitus	PTA	Oral corticosteroids, IM corticosteroids	Partial recovery	Good
10	Pokharel et al. (32)	Nepal	Case report	1	27, M	1 month after COVID-19	Left	HL, Tinnitus	PTA, TFT	Oral corticosteroids	Partial recovery	Fair
11	Edwards et al. (33)	UK	Case report	1	68, F	10 days after COVID-19	Bilaterally	HL	PTA	Oral corticosteroids, IM corticosteroids	Partial recovery	Good
12	Ricciardiello et al. (11)	Italy	Case series	5	26, F	8 days after COVID-19	Left	HL, Tinnitus, Vertigo	PTA, Tympanometry, HST, ABR, THI, DHI	Oral corticosteroids, Oral mesoglycan, HBOT	Partial recovery	Good
					22, M	6 days after COVID-19	Right	HL, Dizziness			Partial recovery	
					61, M	12 days after COVID-19	Left	HL, Tinnitus			Slight improvement	
					30, M	8 days after COVID-19	Bilaterally	HL, Tinnitus			Complete recovery on the right ear, partial recovery on the left ear	
					46, F	6 days after COVID-19	Right	HL, Tinnitus			Partial recovery	
13	Lang et al. (34)	Ireland	Case report	1	30, F	4 weeks after COVID-19	Right	HL, Tinnitus	PTA	Oral corticosteroids	No improvement	Good
14	Rahimi et al. (6)	Iran	Case report	1	60, F	3 days before COVID-19	Left	HL, Tinnitus	PTA, WRS, Tympanometry, TFT	IM corticosteroids	Partial recovery	Good
15	Shah et al. (13)	UK	Case series	4	46, F	3 weeks after COVID-19	Right	HL, Tinnitus, dizziness	PTA, Tympanometry, Telephone consultation	Hearing aid	No improvement	Poor
					43, F	2 weeks after COVID-19	Bilaterally	HL, Vertigo	PTA	NA	NA	
					54, F	A few weeks after COVID-19	Right	HL, Tinnitus	Telephone consultation	Hearing aid	No improvement	
					54, M	8 weeks after COVID-19	Bilaterally	SSNHL, Tinnitus	PTA	Oral corticosteroids	No improvement	
16	Kilic et al. (35)	Turkey	Case series^*^	1	29, M	NA	Right	HL	PTA, Tympanometry, TFT	Oral corticosteroids	Complete recovery	Poor

*COVID-19, 2019 Coronavirus disease; HL, hearing loss; PTA, Pure-tone thresholds; ABR, Auditory brainstem response; ENoG, Electroneuronography; VHIT, Video head impulse testing; WRS, Word recognition scores; TFT, Tuning fork test; OAE, Otoacoustic emissions; VNG, Videonystagmography; HST, Head shaking test; THI, Tinnitus handicap inventory; DHI, Dizziness handicap inventory; IM, Intratympanic; CI, Cochlear implant; HBOT, Hyperbaric oxygen therapy*.

* *Including only one patient with COVID-19-related SSNHL*.

### Incidence

Although case reports of COVID-19-related SSNHL have increased, it remains unknown whether SARS-CoV-2 has contributed to the increased incidence of SSNHL worldwide.

Swain et al. investigated the audiological performance of 472 COVID patients and found that 24 (5.08%) patients had SNHL, most of which were SSNHL (15). Based on self-reported email questionnaires, a retrospective observational study from Denmark showed that over 10% of COVID-19 patients with self-reported chemosensory loss complained of hearing loss (HL) (16). However, the patients included in the study did not receive objective diagnostic and audiological tests, thus affecting the credibility of the study.

Studies from around the world have independently evaluated the impact of the COVID-19 pandemic on the incidence of SSNHL in a single medical center, with inconsistent results. Data from an Italian audiology tertiary referral center showed that the incidence of SSNHL and combined acute cochlear-vestibular involvement was significantly higher during the COVID-19 pandemic than in previous periods, with a more severe clinical presentation of pure-tone audiometry (PTA) (17). A study from Turkey found an increased incidence of SSNHL during the COVID-19 epidemic, with 60.3% of subjects having signs compatible with COVID-19 (18). A large tertiary hospital in China also found an increase in SSNHL visits to the outpatient and emergency departments during the COVID-19 pandemic compared to the past (19). However, several studies found no increase in the incidence of SSNHL after the COVID-19 outbreak, concluding that SARS-CoV-2 does not appear to have a significant risk of SSNHL development (20–23). However, these study data are based on the number of patients who visited the hospital. It is possible that some patients were reluctant to visit the hospital for worry of being infected with COVID-19, thus affecting the accuracy of the incidence of SSNHL (20). Van Rijssen et al. tested 25 patients with SSNHL in a large Dutch teaching hospital for COVID-19 with no positive cases, and concluded no significant relationship between SSNHL and COVID-19 (23). Doweck et al. used data from Clalit Health Services to compare the incidence of SSNHL during the COVID-19 pandemic in Israel with the incidence in 2018 and 2019, and found that the incidence of SSNHL decreased during the COVID-19 pandemic compared with the time before the COVID-19 outbreak (24). It might be related to the decrease in community physicians and emergency department visits (24).

### COVID-19-Related SSNHL

#### Clinical Characteristics

**Figure 2:**
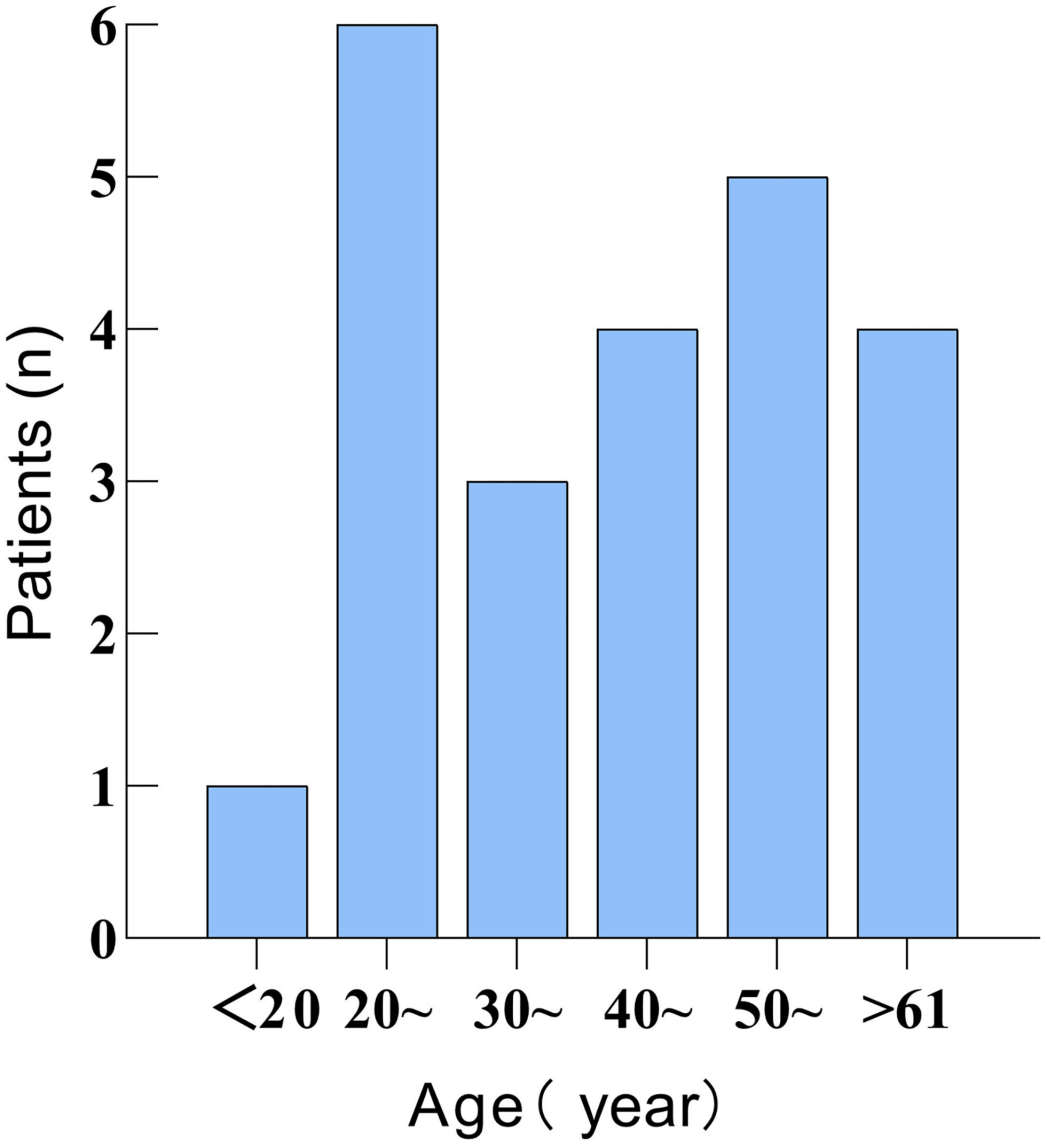
Figure showing the age distribution of 23 patients with 2019 Coronavirus disease (COVID-19)-related sudden sensorineural hearing loss (SSNHL).

There were 23 patients, 11 males and 12 females. The male to female ratio was 0.92. The average age of the patients was 43.1 years (range 18–67 years). The age distribution is shown in Figure 2. Of these patients, six were affected on the left side, eight on the right side, and nine on the bilateral side.

COVID-19- related SSNHL is more common in adults, and audio-vestibular symptoms typically manifest following the diagnosis of COVID-19 or during the rehabilitation period. The time from confirmation of COVID-19 to the onset of SSNHL also varied, which could be anywhere from a few days to 2 months. However, SSNHL in some patients occurred days or 10's of days before confirmation of COVID-19. We speculated that COVID-19 in these patients might have also occurred before SSNHL, confirming COVID-19 somewhat late.

**Figure 3:**
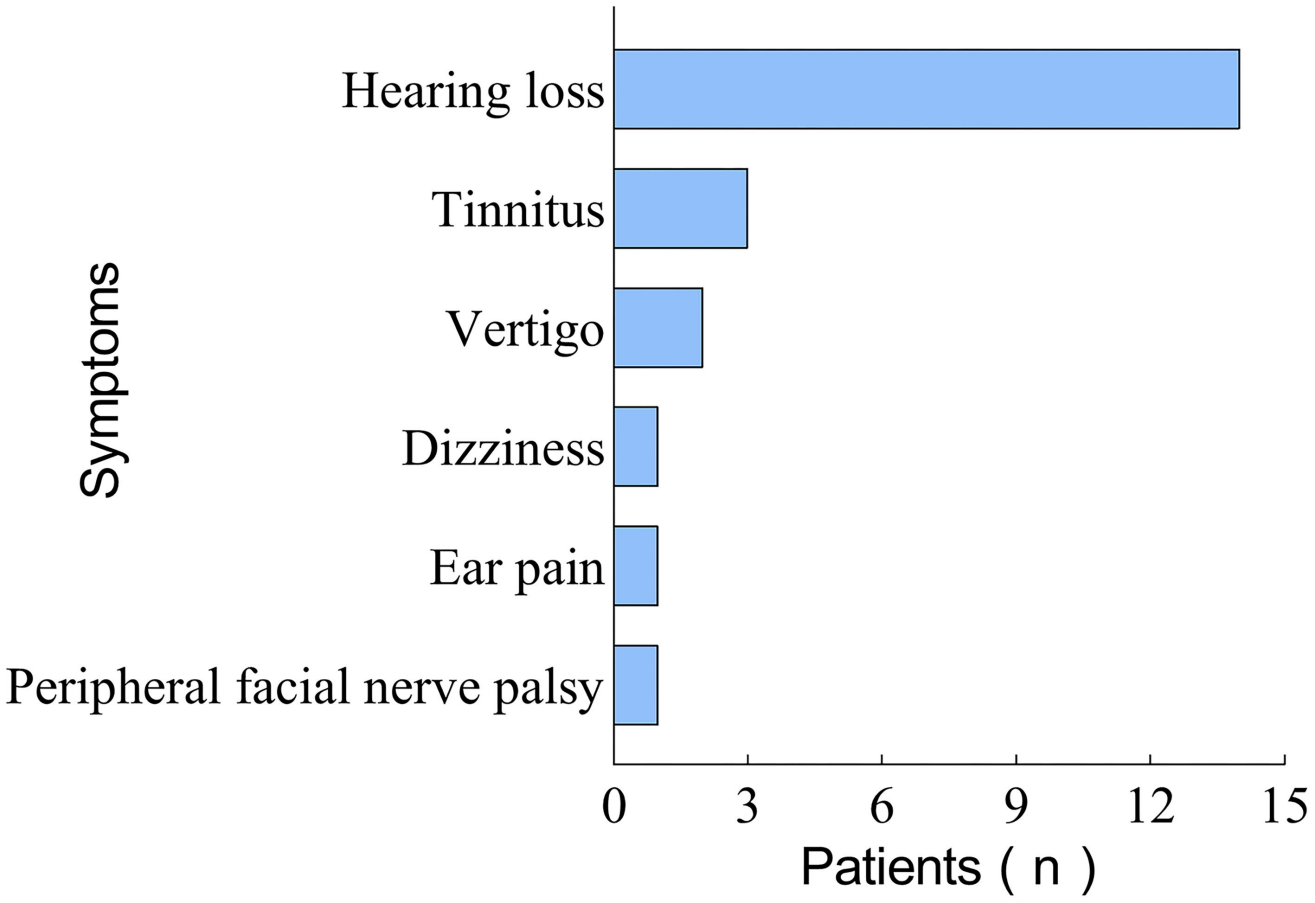
Figure showing the distribution characteristics of concomitant symptoms in 23 patients with COVID-19-related SSNHL.

Of these 23 patients, only 4 (17.4%) presented with HL symptoms solely, while the majority (82.6%) were accompanied by one or more other symptoms. The concomitant symptoms in these patients included tinnitus (*n* = 14, 60.9%), vertigo (*n* = 3, 13.0%), dizziness (*n* = 2, 8.7%), ear pain (*n* = 1, 4.0%), and peripheral facial nerve palsy (*n* = 1, 4.0%). The distribution of accompanying symptoms in patients with COVID-19-related SSNHL is shown in Figure 3. As can be seen, tinnitus is the most common symptom associated with SSNHL.

In most cases, SSNHL is not the first sign of COVID-19 (11). However, there are certain exceptions. Chern et al. described a case of an 18-year-old SSNHL patient who presented to an otology clinic with bilateral auditory fullness and vertigo. The patient was identified with COVID-19 a few weeks later using SARS-CoV-2 IgG antibodies (7). Some individuals have also experienced chemoreceptor dysfunction symptoms, such as anosmia and loss of taste, before developing SSNHL (13, 15).

Most patients with COVID-19-related SSNHL showed normal MRI findings, while some developed an intra-labyrinthine hemorrhage (7, 13). Ozer et al. reported a case of SSNHL with peripheral facial paralysis in a COVID-19 patient, suggesting a link between SSNHL and isolated facial paralysis (25).

#### Audiological Assessments

In these studies, PTA was the most commonly used method of audiological assessment (6, 13, 35). Several studies employed tympanometry and tuning fork tests to identify COVID-19-related SSNHL (6, 35). Due to isolation of patients, telephone consultations were also used to provide an initial assessment of the patient's condition (13). Auditory brainstem response (ABR), otoacoustic emission, and electroneuronography were applied in a few studies (25, 28). Two studies used a head-shaking test or video head impulse testing to identify vertigo (11, 27). The patients' word recognition scores (WRS) were assessed using the speech audiometry method in two studies (6, 7). Also, tinnitus handicap inventory and dizziness handicap inventory were used in one study (11).

#### Treatments

Glucocorticoids were the most commonly used therapeutic agents for COVID-19-related SSNHL, and the vast majority of patients were treated with glucocorticoids. Corticosteroids can be delivered orally, intravenously, or intramuscularly *via* systemic and/or intratympanic (IT) routes (12, 25, 27). In one study, five patients received hyperbaric oxygen therapy (HBOT) and oral mesoglycan as an adjunctive treatment (11). Patients whose hearing did not improve after treatment or who had a history of SSNHL for over 8 weeks were given cochlear implants (CI) and/or hearing aids to enhance their hearing (12, 13, 28). The treatment was not mentioned in the article for one patient (13).

#### Prognosis

**Figure 4:**
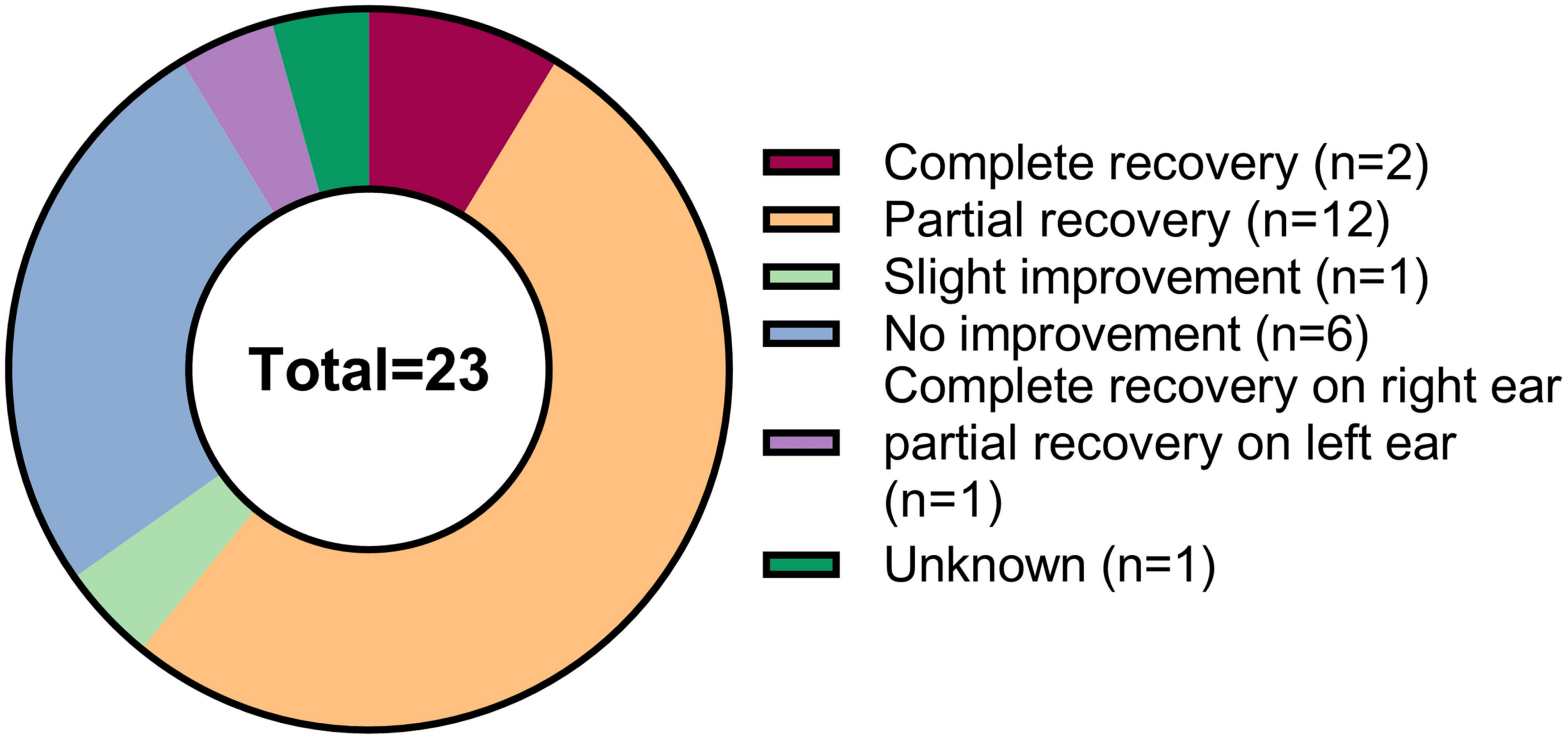
Figure showing the treatment outcomes of 23 patients with COVID-19-related SSNHL.

The cure rate of COVID-19-related SSNHL is unknown since most available studies are case reports or case series with limited sample numbers. Among these 23 cases of COVID-19-related SSNHL, two patients (8.7%) recovered completely, 12 patients (52.2%) recovered partially, 1 (4.3%) case improved slightly, 6 (26.1%) patients did not improve, one patient (4.3%) recovered completely on one side and partially on the other, and one patient (4.3%) with an unknown outcome. The treatment outcomes are presented in Figure 4. Ricciardiello et al. treated and followed up on five individuals with COVID-19-related SSNHL, most of whom recovered partial auditory function (11). Shah et al. described four patients with COVID-19-related SSNHL, two of whom did not improve following treatment with glucocorticoids (13).

## Discussion

This systematic review examined whether COVID-19 leads to an increased incidence of SSNHL by using the available literature. However, the COVID-19 pandemic varied across countries, and the findings were inconsistent and even contradictory. Therefore, it remains unknown whether COVID-19 contributes to the high incidence of SSNHL.

All 23 patients with COVID-19-related SSNHL, included in this systematic review, were adults, with no significant differences in the number of incidences by age group. Pediatric SSNHL is uncommon, and there have been no reports of COVID-19-related SSNHL in children (36). Tinnitus, one of the most common sensorineural disorders, is associated with SSNHL in 66–93% of cases (37). In this review, we found that 60.9% of patients with COVID-19-related SSNHL had tinnitus symptoms similar to the presentation of regular SSNHL.

COVID-19-related SSNHL can occur on one or both sides; however, it is more common on one side (11, 15). Bilateral SSNHL is relatively uncommon (7). However, the likelihood of binaural morbidity in SSNHL was significantly higher in COVID-19 patients than in the general population. Shah et al. studied 4 COVID-19-related SSNHL patients referred to their otolaryngology clinic, 3 of whom presented with bilateral symptoms (13). It may relate to the fact that ototoxicity symmetrically affects both ears (12).

Early audiological assessments and monitoring are critical when suspected or detected with sensorineural hearing loss (SNHL) (38). PTA, otoacoustic emissions, and ABR can all be used to assess the circumstances of patients with COVID-19-related SSNHL (15). PTA helps determine the type and severity of HL. In the era of COVID-19, telemedicine can reduce outpatient follow-up visits, reduce the risk of cross-infection, and improve patient satisfaction (39). According to physical distance requirements and Centers for Disease Control and Prevention guidelines, conventional hearing services are at moderate to high risk for COVID-19 infection (40). As a result, traditional auditory service deliveries are highly restricted. In this context, tele-audiometry has become a workable option for audiometric examination, with a high level of accuracy that is not significantly different from traditional audiometric testing (41). A study by Shilo et al. based on smartphone vibration and uHear App hearing tests also confirmed that the telemedicine model for diagnosing SSNHL is valid and reliable (42). In addition, some improvements can be made to the hearing test technologies to minimize contact with the patient. A recent study has shown that PTA combined with the digits-in-noise test, without bone-conduction thresholds, can distinguish between SNHL and conductive HL (40). Since this hearing test method does not require traditional bone-conduction audiometry in a closed sound booth, it is valuable in preventing cross-contamination.

According to the clinical practice guidelines of the American Academy of Otolaryngology-Head and Neck Surgery, glucocorticoids can be used as initial treatment for SSNHL patients within 2 weeks after the onset of the disease (4). Since there are contradictory results on adverse reactions to corticosteroids in patients with COVID-19, IT administration is a beneficial option (6). A clinical study compared the therapeutic efficacy of IT corticosteroid administration vs. intravenous corticosteroids in COVID-19-related SSNHL, and found comparable efficacy of both treatments (43). However, IT administration did not cause systemic side effects compared to intravenous corticosteroids (43). Therefore, patients with COVID-19-related SSNHL can be treated with IT corticosteroids as soon as they are diagnosed (6).

A recent systematic review and meta-analysis published in JAMA Otolaryngology-Head and Neck Surgery showed, including over 20 years of literature data, that HBOT as part of a comprehensive treatment for patients with SSNHL significantly improves hearing outcomes (44). However, HBOT sessions should be adjusted to meet COVID-19 prevention and control requirements for infection identification, distancing, and isolation, to avoid breathing with an internal chamber atmosphere (45). Moreover, pediatric patients can be safely treated with HBOT (46). Thus, HBOT can be applied while meeting the protection of medical staff and patients to improve the outcomes.

Patients who do not regain their hearing after active treatment may consider hearing aids or CI to improve their hearing. In patients with poor physical condition, CI procedures can be conducted under local anesthesia with analgesia to limit the risk of anesthesia (12).

Individuals with HL may experience challenges, such as difficulty perceiving speech, leading to communication and social difficulties (47). Surgical face masks and face shields were essential for personal protection during the COVID-19 era. However, this impaired the speech perception in individuals with moderate-to-severe HL (48). The pandemic has exacerbated the situation, worsening patients' mental health, particularly in rural areas (49). As a result, the entire society should pay attention to people with HL by giving practical support in various areas such as socialization, detection, treatment, and rehabilitation.

Otolaryngology is considered a high-risk unit for COVID-19 (50). During the ongoing pandemic, there are many infected individuals with atypical symptoms. Each symptom should be considered for association with COVID-19 (51). Kilic et al. detected one positive patient for SARS-CoV-2 among five male patients with unilateral SSNHL as the only complaint in an outpatient otolaryngology clinic (35). SSNHL may be the first symptom of COVID-19. Therefore, all patients with SSNHL and/or acute vestibular disease should be screened for SARS-CoV-2 infection (17, 52). In addition, patients in the otolaryngology outpatient clinic should receive enough attention to their anxiety and stress levels (53).

The majority of the extant research is based on retrospective studies with limited sample sizes, and large-scale prospective investigations are lacking. As a result, large-scale, multicenter research on the pathophysiology, treatment, and prognosis of COVID-19-related SSNHL should be conducted. In the available literature, pure tone audiometry is mainly used to detect COVID-19-related SSNHL, while speech audiometry is rarely used to examine patients' WRS. Speech audiometry may be considered to evaluate the effect of SARS-CoV-2 on cognitive processes.

## Conclusion

SARS-CoV-2 has the potential to cause damage to the audio-vestibular system, resulting in SSNHL. However, the exact mechanisms by which SARS-CoV-2 affects the audio-vestibular system remain unclear. Although numerous investigations have been conducted on COVID-19-related SSNHL, they are fragmented and unsystematic. The true prevalence of SSNHL in COVID-19 patients around the world is unknown. Glucocorticoids are the preferred medication to treat COVID-19-related SSNHL. Hearing testing is recommended when HL is suspected in COVID-19 individuals, and if SSNHL is identified, prompt and vigorous treatment is critical.

## Data Availability Statement

The original contributions presented in the study are included in the article/supplementary material, further inquiries can be directed to the corresponding author.

## Author Contributions

XM and JS conceptualized and drafted the manuscript. JW and KZ critically reviewed the literature and extracted data. XM revised the draft manuscript. All authors contributed to the article and approved the submitted version.

## Funding

This study was funded by the Science and Technology Development Project of the Bureau of Science and Technology of Wuxi, China (Grant number CSZ0N1622).

## Conflict of Interest

The authors declare that the research was conducted in the absence of any commercial or financial relationships that could be construed as a potential conflict of interest.

## Publisher's Note

All claims expressed in this article are solely those of the authors and do not necessarily represent those of their affiliated organizations, or those of the publisher, the editors and the reviewers. Any product that may be evaluated in this article, or claim that may be made by its manufacturer, is not guaranteed or endorsed by the publisher.

## Abbreviations

COVID-19: 2019 Coronavirus disease
SARS-CoV-2: Severe acute respiratory syndrome coronavirus 2
SSNHL: Sudden sensorineural hearing loss
CNS: Central nervous system
ACE2: Angiotensin-converting enzyme 2
TMPRSS2: Transmembrane protease serine subtype 2
ME: Middle ear
IE: Inner ear
HC: Hair cells
MRI: Magnetic resonance imaging
SNHL: Sensorineural hearing loss
WRS: Word recognition scores
PTA: Pure-tone audiometry
HL: hearing loss
HBOT: Hyperbaric oxygen therapy.
