# Corrigendum: COVID-19 and sudden sensorineural hearing loss: a systematic review

**DOI:** 10.3389/fneur.2023.1208650

**Published:** 2023-05-09

**Authors:** Xiangming Meng, Jing Wang, Jian Sun, Kangxu Zhu

**Affiliations:** ^1^Department of Otolaryngology, Wuxi Huishan District People's Hospital, Wuxi, China; ^2^Department of Otolaryngology, Huadong Sanatorium, Wuxi, China; ^3^Department of Rheumatology and Immunology, Affiliated Hospital of Jiangnan University, Wuxi, China

**Keywords:** COVID-19, SARS-CoV-2, sudden sensorineural hearing loss, inner ear, incidence, glucocorticoids

In the published article, there was an error. The word “not” has been missed in one of the sentences in the Conclusion, and for clarity the sentence has been reworded.

A correction has been made to the Conclusion. This sentence previously stated:

“However, the exact mechanisms of SARS-CoV-2 on the audio-vestibular system remain fully understood.”

The corrected sentence appears below:

“However, the exact mechanisms by which SARS-CoV-2 affects the audio-vestibular system remain unclear.”

The authors apologize for this error and state that this does not change the scientific conclusions of the article in any way. The original article has been updated.

